# Index Admission Cholecystectomy for Biliary Colic Precludes the Risk of Readmissions with Biliary Complications and should be Standard Treatment

**DOI:** 10.1007/s00268-022-06847-9

**Published:** 2022-12-16

**Authors:** James Lucocq, Pradeep Patil, John Scollay

**Affiliations:** Department of General and Upper GI Surgery, Ninewells Hospital, Dundee, United Kingdom

## Abstract

**Background:**

Emergency biliary colic admissions can be managed with an index or elective laparoscopic cholecystectomy (LC). Opting to perform an elective LC may have significant repercussions such as the risk of readmissions before operation with further attacks or with biliary complications (e.g. cholecystitis, pancreatitis, choledocholithiasis). The risk of readmission and biliary complications in patients admitted with biliary colic but scheduled for elective surgery has never been investigated. The secondary aim was to compare rates of peri-operative morbidity between the index admission, elective and readmission LC cohorts.

**Method:**

All patients admitted with a diagnosis of biliary colic over a 5-year period and proceeding to LC were included in the study (*n* = 441). The risk of being readmitted and suffering further morbidity whilst awaiting elective LC was investigated. Peri-operative morbidity was compared between the index admission, elective and readmitted LC groups using univariate and multivariate analysis.

**Results:**

Following a biliary colic admission, the risk of readmission whilst awaiting elective LC is significant (2 months-25%; 10 months-48%). In this group, the risks of subsequent biliary complications (18.0%) and the requirement for ERCP (6.5%) were significant. Patients who are readmitted before LC, suffer a more complicated peri-operative course (longer total length of stay, higher post-operative complications, imaging and readmission).

**Discussion:**

Index admission LC for biliary colic avoids the significant risk of readmission and biliary complications before surgery and should be the gold standard. Readmitted patients are likely to have higher rates of peri-operative adverse outcomes. Patients should be counselled about these risks.

## Introduction

Index admission laparoscopic cholecystectomy (LC) reduces the risk of further biliary attacks of biliary colic (BC) [1–3] Nevertheless, patients admitted with BC are frequently discharged with a plan for an elective LC. This subjects them to the risk of subsequent biliary complications (e.g. cholecystitis, pancreatitis, choledocholithiasis) whilst waiting for elective LC, the risk of further intervention (e.g. ERCP), longer length of stay (LOS), as well as other peri-operative adverse outcomes [4–9]. Although the outcomes of index admission versus elective LC for biliary pathology have been compared, the literature mainly focuses on the optimal timing of cholecystectomy for acute cholecystitis. The risks and consequences of readmission before elective LC in patients admitted with BC has not yet been investigated [10–18].

The primary aim of this study was to investigate the risk of readmission and biliary complications in those patients who have been admitted with BC but then scheduled for elective LC. The secondary aim was to compare rates of peri-operative morbidity between the index admission, elective and readmission LC cohorts.

## Methods

### Population cohort

All patients with an index admission of BC who proceeded to a laparoscopic cholecystectomy between January 2015 and January 2020 in one UK health board were included in the study. The health board covers a defined geographical region with a stable population of approximately 493000 people. Operations were performed in one tertiary centre and two day-surgical units under the care of 25 general surgical consultants.

Although an ultrasound scan was the primary radiological investigation of choice on index admission, CT scans were occasionally performed based on clinical severity and MRCP were performed in cases of mild derangement of liver function tests to rule out choledocholithiasis. Any patient with radiological/biochemical evidence of different biliary pathology (e.g. cholecystitis, choledocholithiasis, cholangitis or pancreatitis) were excluded. Therefore, all included patients had normal inflammatory markers and no evidence of inflammation on imaging. Patients attending the Emergency Department with BC and discharged rather than admitted were not included in this study.

### Data collection

Data were collected retrospectively from multiple databases using a deterministic records-linkage methodology. Patients were tracked between databases using a unique 10-digit patient identifier. Data were collected for all pre-operative admissions and then patients were followed-up for 100-days following the operation for all readmissions and outpatient reviews. This data yielded pre-operative data, operative data, significant complications (Clavien-Dindo classification ≥ 2), post-operative imaging, post-operative intervention, post-operative length of stay (PLOS), related readmission data, total length of stay (TLOS) and mortality.

### Risk of readmission

Patients with a BC admission who did not undergo an index admission cholecystectomy were followed up for readmission before cholecystectomy. The rates of biliary complication (e.g. cholecystitis, pancreatitis, choledocholithiasis) and interventions (e.g. ERCP) were evaluated in this group.

The rate of readmission was also displayed using a Kaplan-Meier graph. Cox-proportional hazards model was conducted to determine patient-specific variables that were associated with early readmission.

### Index versus readmission cholecystectomy

**Fig. 1:**
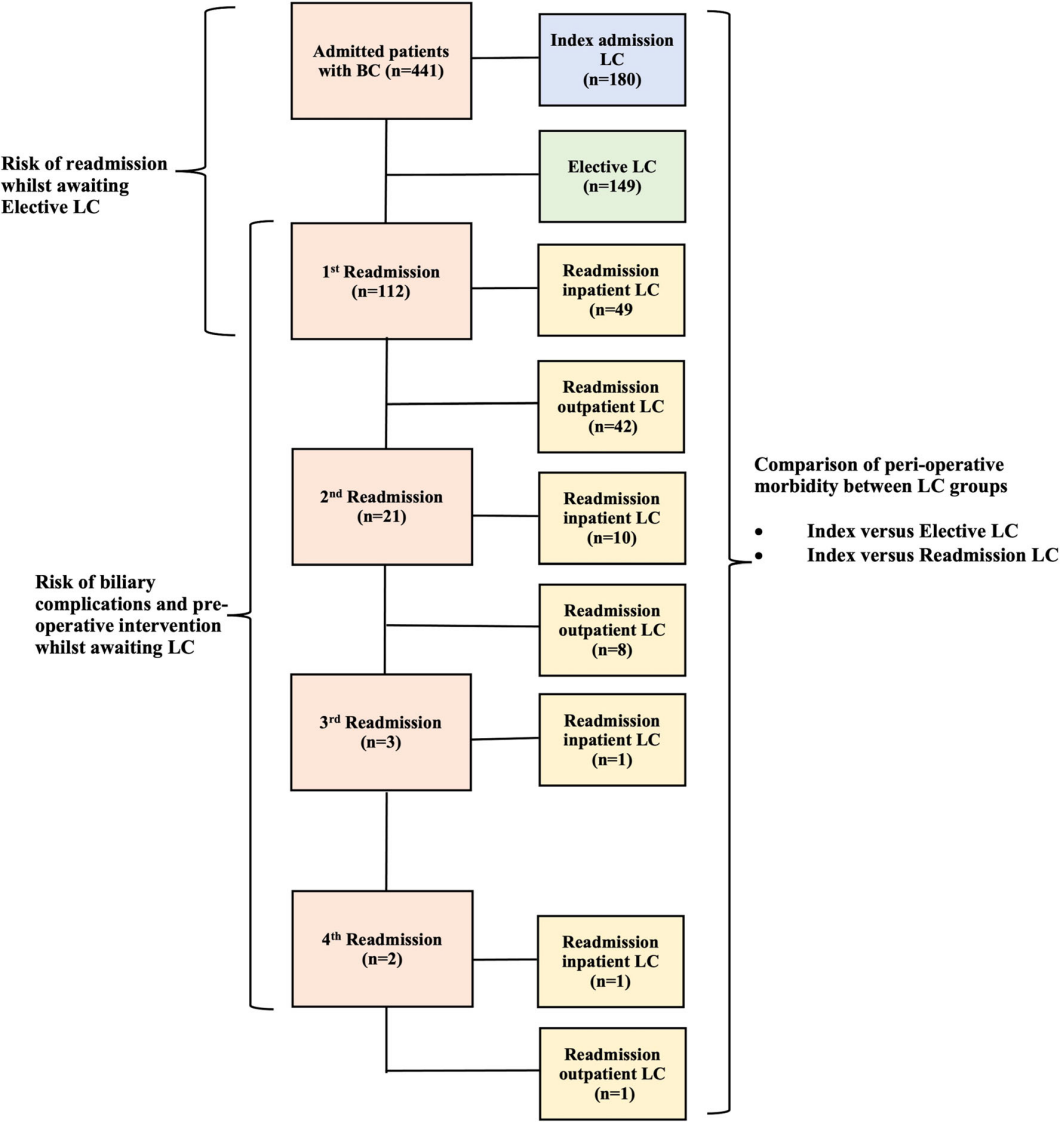
Study design

The peri-operative morbidity of the readmitted group was compared with the index cholecystectomy group (Fig. 1). This was performed using univariate analysis and included Chi-squared, Fischer-exact tests and Mann–Whitney U tests. The following outcome measures were compared: subtotal cholecystectomy; conversion to open; intra-operative complication; drain insertion; post-operative complication/imaging/intervention, length of stay, readmission and mortality.

Multivariate logistic and linear regressions were also conducted to adjust for patient-specific factors: age (< 40; 40–59; ≥ 60 years), gender, ASA score (1; 2; ≥ 3). For each outcome measure (e.g. subtotal, readmission), the most parsimonious model for each adverse outcome was determined by eliminating insignificant variables (*p* > 0.05) using a top-down approach. All statistical tests were conducted using Stata/IC 16.1 statistical software.

### Index versus elective cholecystectomy

The peri-operative morbidity of patients who underwent an index cholecystectomy were compared with those who underwent an elective cholecystectomy (no readmissions) using univariate and multivariate analysis (Fig. 1).

## Results

A total of 441 patients were admitted to hospital with BC. (Table 1). One hundred and eighty patients (40.8%) underwent an index admission LC, 149 patients (33.8%) an elective LC, and 112 patients (25.4%) a LC after readmission. Patients who underwent an index admission LC were more likely to be younger (*p* < 0.001) and had a lower ASA (*p* = 0.001).

**Table 1:** Background data of population cohort

Variable	Patient group				*p*-value (index vs. non-index LC)
	All patients, *n* = 441 (%)	Index LC, *n* = 180 (%)	Elective LC *n* = 149 (%)	Readmission LC, *n* = 112 (%)	
Age, years (%)					< 0.001
< 40	172 (39.0)	86 (47.8)	43 (28.9)	44 (39.3)	
40–59	166 (37.6)	61 (33.9)	66 (44.3)	43 (38.4)	
≥ 60	92 (20.9)	32 (7.3)	40 (26.8)	25 (22.3)	
Male:female	84	1:4.0	1:3.6	1:5.6	0.55
American society of anaesthesiologists score (%)					0.001
1	141 (32.0)	68 (37.8)	41 (27.5)	34 (30.4)	
2	250 (56.7)	99 (55.0)	86 (57.7)	69 (61.6)	
≥ 3	39 (8.8)	12 (6.7)	22 (14.8)	9 (8.0)	
Pre-operative Imaging					
Ultrasound abdomen	425 (96.4)	178 (98.9)	147 (98.7)	110 (98.2)	0.82
CT Abdomen/pelvis	26 (5.9)	9 (5.0)	9 (6.0)	11 (9.8)	0.95
MRCP	155 (35.1)	64 (35.6)	54 (36.2)	56 (50.0)	0.86

### Risk of readmission

Of the patients who did not undergo index admission LC, 42.9% suffered at least one readmission (112/261). Forty-seven patients (18.0%) suffered complications [40 patients (15.3%) cholecystitis, 14 patients (5.4%) choledocholithiasis and 9 patients (3.4%) pancreatitis] and seventeen patients (6.5%) required an ERCP.

**Fig. 2:**
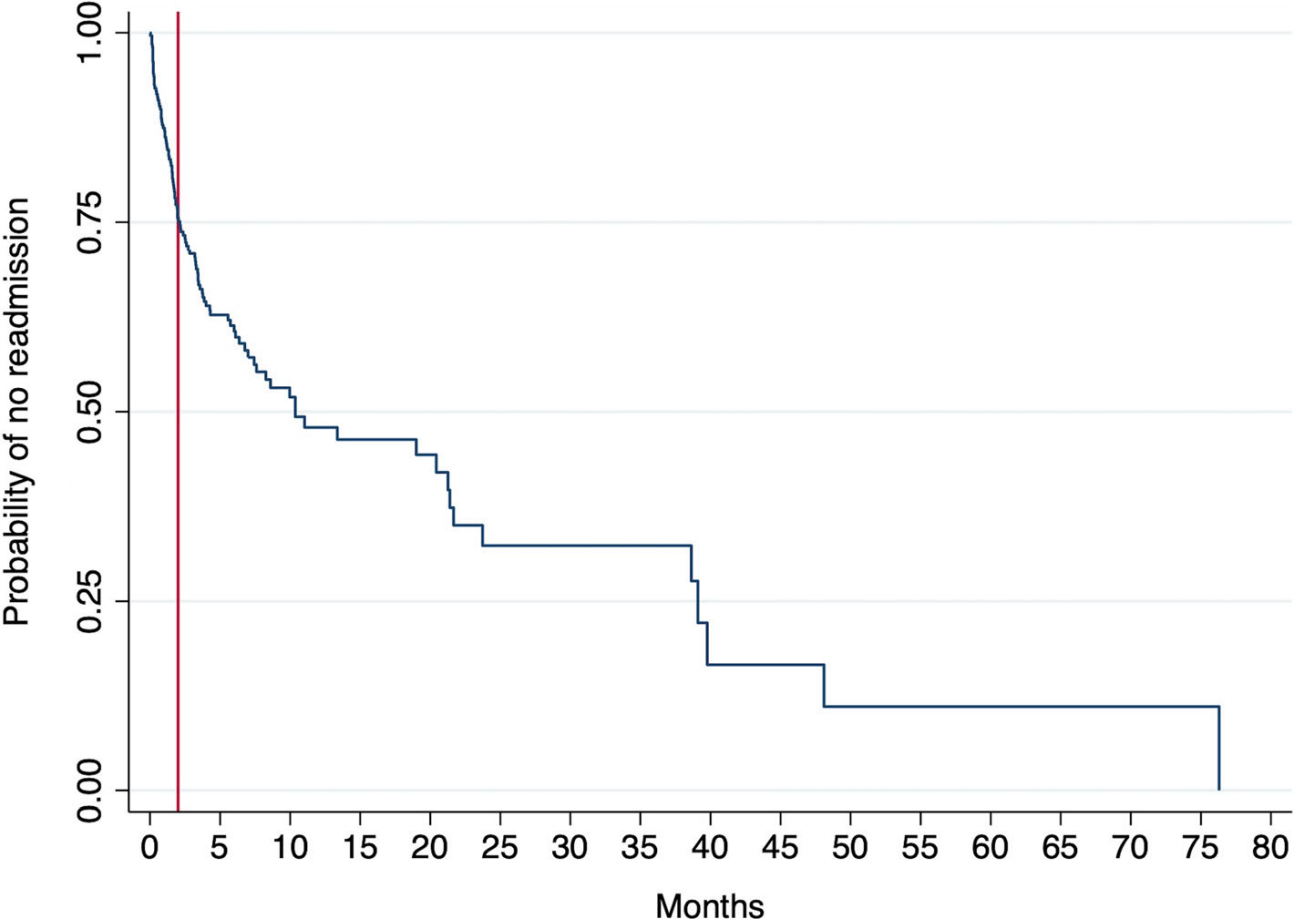
Probability of not being readmitted for elective LC

A Kaplan Meier graph demonstrates a 25% risk of emergency readmission in the first two months following discharge and a 48% risk in the first 10 months (Fig. 2). The median waiting time until elective LC in this cohort was 4 months 12 days at which point the readmission rate was estimated to be 37%. The Cox-proportional hazards model found lower age (age < 40 years) to be the only factor associated with early readmission (HR 1.7, *p* = 0.007; 95% CI 1.16–2.52).

### Index versus readmission LC

The peri-operative outcomes of patients with readmission before cholecystectomy are compared with index cholecystectomy (Table 2). In the univariate analysis, the readmitted patients suffered higher rates of pre-operative intervention (*p* < 0.001), longer TLOS (*p* < 0.001), post-operative imaging (*p* < 0.001) and post-operative readmission (*p* < 0.01). The findings were consistent in the multivariate analysis which demonstrates readmission LC being positively associated with post-operative imaging/intervention, post-operative complication, prolonged post-operative length of stay, post-operative readmission, *p* < 0.05 (Table 3). This adjusted for patient age, gender and comorbidities.

**Table 2:** Peri-operative outcomes of index, elective and readmission LC

Variable	Patient group				Elective versus Index, RR (95% CI)	Readmission versus Index, RR (95% CI)
	All patients	Index LC, *n* = 180 (%)	Elective LC, *n* = 149 (%)	Readmission LC, *n* = 112 (%)		
Pre-operative ERCP	17 (3.9)	0 (0.0)	0 (0.0)	17 (15.2)	–	–***
Intra-operative complication	6 (1.4)	1 (0.6)	3 (2.0)	2 (1.8)	3.6 (0.38–34.48)	3.2 (0.29–35.04)
Bile leak*	3 (0.7)	0 (0.0)	1 (0.7)	2 (1.8)	–	–
Haemorrhage	3 (0.7)	0 (0.0)	3 (2.0)	0 (0.0)	–	–
Bile duct injury	0 (0.0)	0 (0.0)	0 (0.0)	0 (0.0)	–	–
Other	3 (0.7)	1 (0.6)	2 (1.3)	0 (0.0)	2.4 (0.22–26.39)	–
Median time from admission to operation, days (range)	46 (0–2497)	0 (0–1)	113 (6–2497)	133 (5–1505)	–	–
Median operation time, minutes, (range)	72	70	68	75	–	–
Subtotal Cholecystectomy	6 (1.4)	2 (1.1)	2 (1.3)	2 (1.8)	1.2 (0.17–8.47)	1.6 (0.23–11.25)
Conversion to open	1 (0.2)	0 (0.0)	0 (0.0)	1 (0.9)	–	–
Intra-operative drains	14 (3.2)	4 (2.2)	5 (3.4)	5 (4.5)	1.5 (0.41–5.52)	2.0 (0.55–7.32)
Post-operative complication (Clavien ≥ 2)	28 ()	11 (6.1)	7 (4.7)	10 (8.9)	0.8 (0.31–1.93)	1.5 (0.64–3.33)
Bile leak	5 (1.1)	2 (1.1)	1 (0.7)	2 (1.8)	0.6 (0.05–6.60)	1.6 (0.23–11.25)
Collection	10 (2.3)	4 (2.2)	3 (2.0)	3 (2.7)	0.9 (0.21–3.98)	1.2 (0.27–5.29)
Retained stone	5 (1.1)	1 (0.6)	1 (0.7)	3 (2.7)	1.2 (0.08–19.15)	4.4 (0.51–45.79)
Other	11 (2.5)	5 (2.8)	3 (2.0)	3 (2.7)	0.7 (0.18–2.98)	1.0 (0.24–3.96)
Median TLOS, days (range)	4 (0–47)	4 (0–22)	4 (2–15)	8 (2–47)	–	–***
Median PLOS, days (range)	1 (0–19)	1 (0–16)	1 (0–10)	1 (0–19)	–	–
Post-operative imaging and intervention						
Imaging	58 (13.2)	17 (9.4)	14 (9.4)	27 (24.1)	1.0 (0.51–1.95)	2.6 (1.46–4.46) ***
ERCP	8 (1.8)	3 (1.7)	1 (0.7)	4 (3.6)	0.4 (0.04–3.81)	2.1 (0.49–9.40)
Return to theatre	3 (0.7)	0 (0.0)	2 (1.3)	1 (0.9)	–	–
Radiological drainage	1 (0.2)	1 (0.6)	0 (0.0)	0 (0.0)	–	–
Death	0 (0.0)	0 (0.0)	0 (0.0)	0 (0.0)	–	–
Rate of Readmission	44 (10.0)	14 (7.8)	11 (7.4)	19 (17.0)	0.9 (0.44–2.03)	2.2 (1.14–4.17)*

*Statistical significance *p* < 0.05; **p < 0.01; ***p < 0.001

**Table 3:** Comparison of outcomes between index admission and readmission laparoscopic cholecystectomy, multivariate logistic regression

Outcome*	OR	Std. Err	*p*-value	95% CI
Intraoperative complication	4.92	6.50	0.22	0.37–65.79
Subtotal	3.30	4.18	0.28	0.28–39.56
Post-operative complication	2.73	1.29	0.03	1.09–6.88
Prolonged post-operative stay	2.72	1.15	0.02	1.19–6.21
Post-operative imaging or intervention	3.11	1.03	0.001	1.63–5.94
Readmission	2.50	0.98	0.01	1.22–5.43

*Conversion to open rate was 0% in the index admission group and therefore the OR was ill-defined

### Index versus Elective LC

In both univariate and multivariate analysis, there was no significant difference in any adverse outcome between index and elective LC, *p* > 0.05 (Tables 2, 4).

**Table 4:** Comparison of outcomes between index admission and elective laparoscopic cholecystectomy, multivariate logistic regression

Outcome*	OR	Std. Err	*p*-value	95% CI
Intraoperative complication	2.94	3.47	0.37	0.29–29.85
Subtotal cholecystectomy	1.82	2.30	0.64	0.15–21.6
Post-operative complication	1.07	0.63	0.90	0.34–3.34
Prolonged post-operative stay	0.67	0.37	0.46	0.23–1.96
Post-operative imaging or intervention	1.09	0.41	0.814	0.52–2.29
Readmission	1.03	0.45	0.94	0.44–2.41

*Conversion to open rate was 0% in the index admission group and therefore the OR was ill-defined

## Discussion

This study demonstrates index admission LC to be the superior mode of treatment. Although both index admission and elective cholecystectomy are seen as acceptable modes of treatment, discharging a patient to await an elective LC subjects them to significant risk of readmission (25% in 2 months) and subsequent biliary complications (18.0%). If readmitted, the peri-operative course is considerably more complicated (*p* < 0.05) and the overall hospital stay increases significantly (by an average of 4 days).

The risk of developing biliary complications in BC patients appears to correlate with severity of symptoms. Patients with mild symptoms, not requiring admission, reportedly develop complications at a rate of 1–3% annually, whereas those attending an emergency department re-attend in up to 40.9% of cases before cholecystectomy [4, 5]. The readmission rate in patients admitted with BC has not previously been reported, but was found to be significant (42.9%) and demonstrates the notable rate of complications in patients that have severe symptoms requiring admission. Index admission obviates this risk through early surgery and should be considered the gold standard of treatment [6–9]. Although emergency department attendances were not acknowledged in this study, this patient group, similar to the admitted cohort may also benefit from early cholecystectomy [5] and should be included in future studies.

Despite the main finding of this study, there may be cases where index admission cholecystectomy is not appropriate. For example, patients on anticoagulants may be better managed through an elective pathway [19]. Patients awaiting optimisation of medical conditions (e.g. COPD, heart failure) may also benefit from medical review or anesthetic assessment prior to surgery [20]. Although elective cholecystectomy may be preferable in such patients it can be suggested that, those who have been hospitalised should be prioritised ahead of those who have not required an admission as they are at a higher risk of readmission and complication. In these deferred patients, aiming to perform a timely elective cholecystectomy within 2 weeks of discharge, similar to gallstone pancreatitis, should be the recommendation [21].

The findings of this study have significant implications for resource utilisation. Healthcare services are under significant strain to deliver timely elective cholecystectomy, particularly in western populations where the burden of gallstone disease is high. This often results in long waiting times [17]. If patients admitted with BC are added to a lengthy waiting list for elective surgery they are exposed to a significant risk of readmission, biliary complications and increased peri-operative morbidity. Therefore, index admission surgery must be advocated as a cost-effective strategy for BC patients as this will reduce readmissions, overall morbidity and waiting times. This issue is ever more pertinent in the light of the COVID-19 pandemic which has resulted in increasing waiting list times and the further exposure to the risk of readmission [18, 22–24].

The principal studies comparing index admission and elective cholecystectomy in patients admitted with biliary pathology do not acknowledge readmission morbidity before the operation and its impact on overall outcome [10–18]. This study demonstrates that the morbidity associated with readmission in BC patients is more severe than the difference in morbidity between index and elective cholecystectomy and cannot be disregarded. The same finding may be true for other biliary pathology (e.g. cholecystitis) and must be considered in the future analyses [25].

Interestingly, this study suggests that younger and less comorbid patients are more likely to undergo index admission cholecystectomy. It could be postulated that on call surgeons prefer to operate on fitter, healthier patients as they perceive such individuals as more straightforward. However, this approach may be counter-intuitive as it exposes more vulnerable patients to the risk of readmission, biliary complications, longer length of stay and associated peri-operative morbidity. Prioritisation of index admission cholecystectomy in older more comorbid patients could help mitigate this risk and reduce overall morbidity. Clearly this argument does not hold in the small cohort taking anticoagulant medications or in need of medical optimisation prior to surgery.

In summary there is considerable demand for index admission cholecystectomy. Resource issues will often mean that not every patient with gallstone disease can be operated on during their initial presentation. It has not yet been determined which particular biliary conditions (e.g. biliary colic, acute cholecystitis, choledocholithiasis) benefit most from index admission cholecystectomy and should be prioritised by an on call team. Many studies suggest that early laparoscopic cholecystectomy is the optimal treatment for patients with acute cholecystitis. Nevertheless, a proportion of such patients will present late in their illness, potentially out with the perceived window for safe surgery [10–13, 26]. In cases of gallstone pancreatitis, index admission LC has also been proposed as the optimal treatment option due to a reduction complications compared to delayed LC [27]. The present study suggests that biliary colic admissions should be managed as complicated gallstone disease and also undergo an index admission LC.

Hence, the BC patient may be an ideal surgical candidate that should be prioritised for index admission LC, as by definition there is no gallbladder inflammation and the suspicion of ductal stones is low [23, 24, 28]. This approach can be considered a low-risk strategy which reduces overall morbidity as well as the rate of biliary readmissions. Emergency biliary presentations consume a huge amount of resource and biliary readmission are an unfortunate and illogical consequence of busy healthcare systems. Whilst the results of this study cannot be regarded as conclusive evidence that BC patients should be prioritised over other sub-groups, it must be worthy of consideration. Further work is required to determine exactly which sub-groups of patients benefit most from index admissions LC as they should be prioritised by an on-call team.

Whilst index admission LC is the gold standard mode of treatment for BC, it is clear that not all of these patients will receive index admission LC. The present study only includes patients who ultimately proceeded to LC, thus it may overestimate the readmission and complication rate whilst awaiting LC. From our experience the vast majority of those admitted with biliary colic proceed to LC and this isn’t anticipated to significantly influence the results. Regardless of the rate of readmission, the additional risk associated with LC following readmission is demonstrated and index admission LC is the safest option. Indeed these patients should be managed differently to those patients who have simply been referred to a surgical clinic, with expedited surgery. If such patients are unable to undergo LC due to limited resources during their index admission they should be discharged with a scheduled imminent date for surgery. Such an approach will ultimately reduce cost, morbidity and reduce bed occupancy.

Of course adoption of this approach is subject to recourses such as inpatient bed capacity, access to emergency theatre and on-call staffing. Regardless, recognition of index admission cholecystectomy as the gold standard treatment option should be appreciated and on-call surgeons should strive to offer this where possible.

## Conclusion

Index admission laparoscopic cholecystectomy for biliary colic avoids the significant risk of readmission and biliary complications before surgery and should be the gold standard. Readmitted patients are likely to have higher rates of peri-operative adverse outcomes. Patients should be counselled about these risks.

## Data Availability

Data will be available on request.
